# Continuation, Resumption, and Withdrawal Rates of CGRP-mAb Treatment for Migraine Under Real-World Clinical Conditions in Which Patients Are Free to Choose Own Treatment

**DOI:** 10.3390/neurolint18010003

**Published:** 2025-12-24

**Authors:** Takafumi Tanei, Satoshi Yamashita, Satoshi Maesawa, Yusuke Nishimura, Tomotaka Ishizaki, Yoshitaka Nagashima, Takahiro Suzuki, Hajime Hamasaki, Shun Yamamoto, Toshihiko Wakabayashi, Ryuta Saito

**Affiliations:** 1 Department of Neurosurgery, Graduate School of Medicine, Nagoya University, Nagoya 466-8550, Japan; yusukenishimura0411@gmail.com (Y.N.); saito.ryuta.b1@f.mail.nagoya-u.ac.jp (R.S.); 2 Department of Specialized Headache Outpatient, Nagoya Garden Clinic, Nagoya 451-0051, Japan; 3 Department of Neurosurgery, National Hospital Organization, Nagoya Medical Center, Nagoya 460-0001, Japan

**Keywords:** CGRP, continuation, migraine, resumption, withdrawal

## Abstract

Background/Objectives: Anti-calcitonin gene-related peptide monoclonal antibodies (CGRP-mAbs) are effective injectable medications for the treatment of migraine. This study aimed to evaluate continuation, resumption, and withdrawal rates of CGRP-mAb treatment under real-world clinical conditions. Methods: Treatment-naïve patients with at least 3 months of follow-up after starting CGRP-mAb treatment were included. The decision to continue, discontinue, or resume CGRP-mAb treatment was made freely by the patients. Headache Impact Test-6 (HIT-6) and the Migraine-Specific Quality of Life Questionnaire (MSQ) were administered before starting treatment and one month after each CGRP-mAb injection. The endpoints were as follows: continuation rates of CGRP-mAb treatment after treatment initiation; resumption rate; withdrawal rate; changes in HIT-6 and MSQ scores; and differences in background factors between the resumption and withdrawal groups. Results: Of the 1162 migraine patients, 146 were included in the analysis. Continuation rates of CGRP-mAb treatment at 3, 6, 9, 12, 18, and 24 months were 93.2%, 80.2%, 68.9%, 58.8%, 55.4%, and 51.7%, respectively. For the patients who discontinued, the resumption rate was 76.8%, and the withdrawal rate was 20.7%. HIT-6 and MSQ scores were significantly decreased at all assessment points compared with before CGRP-mAb treatment. There were no significant differences in factors between the resumption and withdrawal groups. Conclusions: Under real-world clinical conditions in which patients were free to choose their own treatment, the continuation rate of CGRP-mAb treatment 12 months after treatment initiation was 58.8%, and more than half of patients remained on treatment after 24 months. The resumption rate was 76.8% and the withdrawal rate was 20.7%.

## 1. Introduction

Migraine is a common neurological disorder that affects over one billion people worldwide, not only reducing the quality of life of patients, but also causing significant economic losses [1,2]. Migraine attacks can cause severe headaches and vomiting, leading to missed work or school (known as absenteeism), whereas mild headaches may not be severe enough to cause missed work or school, but may still prevent optimal performance and reduce productivity (known as presenteeism) [2,3]. According to the Global Burden of Disease Study, the top five diseases and disabilities that contributed most to years lived with disability were low back pain, migraine, age-related and other hearing loss, iron-deficiency anemia, and major depressive disorder. Migraine was ranked second in both 1990 and 2016 [4]. In Japan, the annual economic losses associated with absenteeism and presenteeism due to migraine were estimated to be USD 2.7 billion and USD 21.3 billion, respectively [3]. Furthermore, even patients with infrequent headaches have been found to experience substantial disability, interictal burden, and impact on productivity and quality of life [5]. Presenteeism due to migraine was found to be a more serious problem as a cause of work loss than absenteeism. Migraine is a curable disease, and, therefore, appropriate treatment can reduce the impairment of patients’ quality of daily life and reduce social and economic losses.

Medical treatments for migraine are divided into two categories: acute medications that suppress migraine attacks, and prophylactic medications that reduce the frequency and intensity of headaches [2]. Conventional acute medications include triptans, acetaminophen, and nonsteroidal anti-inflammatory drugs. A new acute medication, lasmiditan, has become available in Japan, and its efficacy in the real-world setting has been reported [6]. Prophylactic medications are available in two forms: oral and injectable. Oral prophylactic medications include anticonvulsants, antidepressants, calcium channel blockers, and beta-blockers. The recently developed injectable anti-calcitonin gene-related peptide monoclonal antibodies (CGRP-mAbs) have dramatically improved the preventive treatment of migraine [7,8,9,10,11]. Three CGRP-mAbs, galcanezumab (GAL), fremanezumab (FRE), and erenumab (ERE), are available in Japan. CGRP-mAb treatment significantly reduces migraine attack frequency and headache intensity [12]. Furthermore, even in difficult-to-treat migraine with medication overuse headache (MOH), CGRP-mAbs may reduce headache frequency and acute medication use without the need for abrupt medication discontinuation or hospitalization [13]. Although these effective new medications are now available for migraine, appropriate migraine treatment is still not widely available to migraine patients in Japan, with only 14.8% using triptans and only 9.2% using oral prophylactic medications [14]. Thus, the initiation rate of CGRP-mAb treatment is estimated to be lower than the use rate of oral prophylactic medications.

In 2022, guidelines were updated to recommend that migraine patients continue CGRP-mAb treatment for 12 to 18 months after initiation, since symptoms worsen if treatment is discontinued [15,16,17,18]. However, CGRP-mAb treatment is expensive and requires monthly injections, which imposes a financial and physical burden on patients who continue this treatment for longer than 12 months. In real-world clinical practice, decisions to initiate, continue, discontinue, or resume CGRP-mAb treatment are primarily based on patient preference, which may not follow guideline recommendations. In Japan, the condition for initiating CGRP-mAb treatment is that patients are taking at least one oral prophylactic medication, but there are no restrictions on discontinuing or resuming the treatment. Only a few studies have reported continuation, discontinuation, and resumption of CGRP-mAb treatment in real-world clinical practice [19,20,21].

Therefore, the aim of the present study was to evaluate the time course of continuation rates of CGRP-mAb treatment for migraine, and rates of resumption and withdrawal after CGRP-mAb discontinuation under real-world clinical conditions in which patients are free to choose own treatment.

## 2. Materials and Methods

### 2.1. Study Design

This was a single-center, retrospective, observational study of patients with migraine who received CGRP-mAb treatment. The patients were recruited from the specialized headache outpatient clinic at Nagoya Garden Clinic from May 2022 to August 2025. All patients had a diagnosis of migraine according to the International Classification of Headache Disorders 3 criteria [22]. Patients first underwent magnetic resonance imaging to exclude intracranial diseases, and then medical treatment for migraine was started. Diagnosis and treatment were performed by a headache specialist and neurosurgeon (T.T.). In cases with frequent headaches, oral prophylactic medications such as anticonvulsants, antidepressants, calcium channel blockers, and beta-blockers were prescribed based on the attending physician’s experience. Oral prophylactic medicines were given for at least 2 months. CGRP-mAb treatment was started when the frequency of headaches or migraines did not decrease to the patient’s satisfaction even after receiving one or more oral prophylactic medications, and the patients agreed to the treatment. The selection of CGRP-mAb was not random, but one of three CGRP-mAbs (GAL, FRE, ERE) was selected through patient-physician discussion in clinical practice. The decision to continue, discontinue, or resume CGRP-mAb treatment was based on patient preference. If the selected CGRP-mAb was insufficient or became less effective, it was switched to another CGRP-mAb according to patient preference. Quarterly FRE was administered if the patient preferred it. Patients included in the analysis were CGRP-mAb treatment-naïve patients with at least 3 months of follow-up after the initial CGRP-mAb administration. This study was approved by the Ethics Review Committee of Nagoya University Graduate School of Medicine (approval number 2022-0316). Since this study was noninvasive, the Ethics Review Committee of Nagoya University Graduate School of Medicine waived the requirement for written, informed consent from patients, but the opt-out method was adopted in accordance with the Japanese ethics guidelines. This research was completed in accordance with the Declaration of Helsinki as revised in 2013.

Inclusion criteria

- CGRP-mAb naïve
- Follow-up ≥ 3 months after initial CGRP-mAb
- If the above conditions were met, the following cases were included
  - Patients who switched CGRP-mAb within the first 3 months
  - Discontinued CGRP-mAb due to adverse events or others
  - Others

Exclusion criteria

- CGRP-mAb non-naïve
- Lost < 3 months after initial CGRP-mAb
- Follow-up < 3 months after initial CGRP-mAb

### 2.2. Data Collection

Demographic data (age, sex, onset years of migraine, family history of headache, history of psychiatric disorders, migraine with aura, smoking, precipitating factors, classification of migraines, headache questionnaire scores, use of oral prophylactic medications and type, type of initial CGRP-mAb, and timings of initiation of CGRP-mAb) were collected retrospectively. Migraine was classified into four types: episodic migraine (EM), high-frequency episodic migraine (HFEM), chronic migraine (CM), and MOH. Monthly headache days of 0–7 days were defined as EM, and 8–14 days as HFEM. The Headache Impact Test-6 (HIT-6) and Migraine-Specific Quality of Life Questionnaire (MSQ) were administered before starting CGRP-mAb treatment (baseline) and 1 month after each CGRP-mAb injection. The timing of initiation of CGRP-mAbs was categorized as follows: early introduction, after trying one first oral prophylactic medication; and late introduction, after trying two or more prophylactic medications. Patients were free to discontinue or resume CGRP-mAb treatment at any time and for any reason. Continuation, resumption, and withdrawal of CGRP-mAb treatment were defined as follows. Continuation was defined as the following three patterns: continued the same CGRP-mAb without interruption, continued even if the type of CGRP-mAb was switched, and treatment was temporarily discontinued because CGRP-mAb was effective, but it was resumed within 3 months of discontinuation. Resumption was defined as when CGRP-mAb treatment was discontinued due to its efficacy, but it was then resumed within 12 months. Withdrawal was defined as meeting the following two conditions: CGRP-mAb treatment was discontinued because it was effective, and the patient did not wish to resume treatment more than three months after discontinuation.

Definition

- Continuation
  - Continued the same CGRP-mAb
  - Continued CGRP-mAbs even after switching to different types
  - CGRP-mAb was effective, discontinued, resumed within 3 months

- Resumption
  - CGRP-mAb was effective, discontinued, resumed within 12 months

- Withdrawal
  - CGRP-mAb was effective, discontinued, no resume more than 3 months

### 2.3. Assessments and Statistical Analysis

The primary endpoint of this study was the continuation rate of CGRP-mAb treatment 12 months after treatment initiation. The secondary endpoints were examined for all patients included in the analysis: first, continuation rates of CGRP-mAb treatment at 3, 6, 9, 18, and 24 months after treatment initiation; second, the resumption rate, timing of discontinuation, interval from discontinuation to resumption, and the withdrawal rate; third, changes in HIT-6 and MSQ scores from baseline, 3, 6, 9, 12, 18, and 24 months after CGRP-mAb treatment initiation; and fourth, in patients who discontinued CGRP-mAb treatment because it was effective, differences in background factors were examined between the group that resumed CGRP-mAb treatment (Resumption group) and the group that was able to withdraw (Withdrawal group).

The Shapiro–Wilk test was used to check whether each variable followed a normal distribution. Next, for categorical variables, the Wilcoxon signed-rank test was performed for headache questionnaire scores. The Mann–Whitney U test was used to compare the resumption and withdrawal groups. Fisher’s exact test was used to evaluate categorical variables for items with a small sample size. The Bonferroni method was used to adjust for multiple comparisons and correct for repeated measurements. Significance was set at *p* < 0.05. Statistical power was calculated for each item. All statistical analyses were performed using EZR (Saitama Medical Center, Jichi Medical University, Saitama, Japan), a graphical user interface for R (The R Foundation for Statistical Computing, Vienna, Austria), a modified version of R commander designed to add statistical functions frequently used in biostatistics [23].

## 3. Results

### 3.1. Participants’ Demographic Characteristics

**Figure 1:**
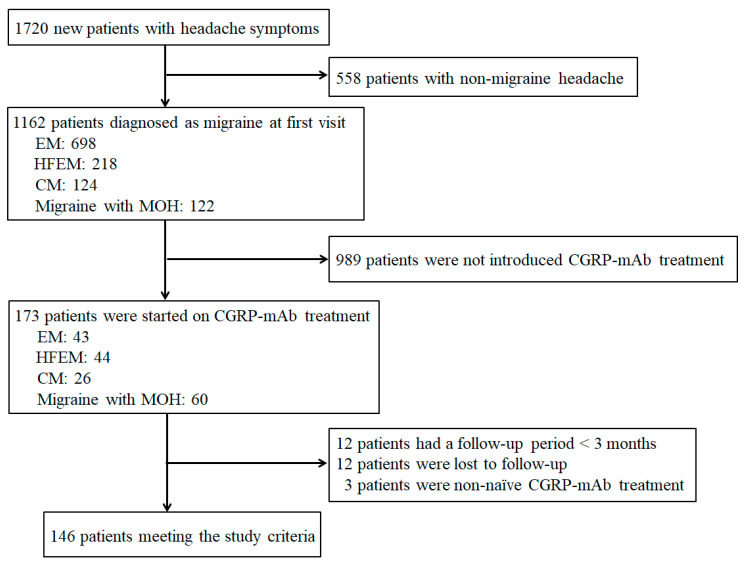
Flowchart showing patient selection.

From May 2022 to August 2025, 1720 new patients with headache symptoms visited the specialized headache outpatient clinic, of whom 1162 (67.6%) were diagnosed as having migraine. Migraine classifications for all patients during the study period were EM (*n* = 698, 60.1%), HFEM (*n* = 218, 18.8%), CM (*n* = 124, 10.7%), and migraine with MOH (*n* = 122, 10.5%). Of all patients with migraine, 173 (14.9%) were receiving CGRP-mAb treatment, including EM (*n* = 43, 24.9%), HFEM (*n* = 44, 25.4%), CM (*n* = 26, 15.0%), and migraine with MOH (*n* = 60, 34.7%). Twenty-seven patients who received CGRP-mAb treatment were excluded from the analysis due to follow-up duration less than 3 months (*n* = 12), lost to follow-up within 3 months after initial treatment (*n* = 12), or non-naïve CGRP-mAb treatment (*n* = 3). Therefore, 146 patients were included in the analysis (Figure 1).

The mean follow-up period from the initial CGRP-mAb administration was 15.3 ± 9.5 months. The clinical characteristics of the patients included in the analysis are shown in Table 1. The mean age was 36.8 ± 12.8 years, with females accounting for 83.6% of patients (122/146). The onset ages of migraine were teens and younger (*n* = 88, 60.3%), 20s (*n* = 44, 30.1%), 30s (*n* = 13, 8.9%), and 40s and older (*n* = 1, 0.7%). Ninety-five patients had a family history of headaches (65.1%), 20 patients had a history of psychiatric disorders (13.7%), 70 patients had migraine with aura (47.9%), and 7 patients had a smoking history (4.8%). The precipitating factors (including overlaps) were weather (*n* = 109, 74.7%), temperature (*n* = 47, 32.2%), alcohol (*n* = 21, 14.4%), and menstruation (*n* = 60, 41.1%). The migraine classifications of the patients were EM (*n* = 31, 21.2%), HFEM (*n* = 37, 25.3%), CM (*n* = 26, 17.8%), and migraine with MOH (*n* = 52, 35.6%). At baseline before CGRP-mAb treatment, the median [interquartile range; IQR] HIT-6 and MSQ scores were 62.0 [60.0–65.0] and 34.0 [27.0–43.0], respectively. The types and number of oral prophylactic medications (including overlaps) used in analyzed patients included antiepileptics (132), antidepressants (81), calcium channel blockers (59), and beta-blockers (7). The CGRP-mAbs initially selected were GAL (55), FRE (45), and ERE (46). The timings of initiation of CGRP-mAb were as follows: early introduction (*n* = 63, 43.2%) and late introduction (*n* = 83, 56.8%).

A total of 1787 CGRP-mAbs were administered during the study period: GAL (766), FRE (558), and ERE (463). Of these, 62 CGRP-mAbs administered to 27 excluded patients were excluded from the analysis. Therefore, 1725 CGRP-mAbs administered to the 146 patients included in the analysis were analyzed (Table 2). Of the 102 patients who continued initial CGRP-mAb, 44 switched at least once during the study period; 25 patients switched once, 12 twice, and 7 three times. Some patients switched multiple times, resulting in a total of 70 switches among the 40 patients who switched. The reasons for switching CGRP-mAbs (decreased efficacy, side effects, cost, others), the number of doses after switching, and the time to the first switch were shown in Table 2. In patients who switched to another CGRP-mAb because of a decrease in efficacy of one, HIT-6 and MSQ scores before and after switching were 60.0 [58.0–64.0] and 32.0 [25.0–38.5], and 59.0 [54.8–60.0] and 26.0 [19.8–35.0], respectively.

### 3.2. Continuation Rates of CGRP-mAb Treatment

**Figure 2:**
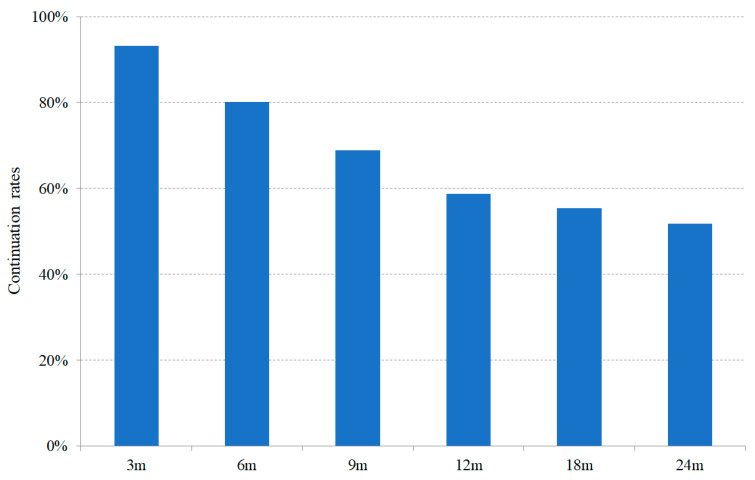
The time course of continuation rates of CGRP-mAb treatment.

The numbers of patients with follow-up periods of 3, 6, 9, 12, 18, and 24 months after starting CGRP-mAb treatment were 146, 126, 103, 80, 56, and 29, respectively. The numbers of patients continuing CGRP-mAb treatment at the same time points were 136, 101, 71, 47, 31, and 15, respectively. Therefore, the continuation rate of CGRP-mAb treatment at 12 months, the primary endpoint, was 58.8% (47/80). Of these 47 patients, 15 continued receiving the same CGRP-mAb, 12 continued receiving a CGRP-mAb after switching the type, and 20 temporarily discontinued but then resumed CGRP-mAb treatment. Continuation rates of CGRP-mAb treatment at 3, 6, 9, 18, and 24 months were 93.2%, 80.2%, 68.9%, 55.4%, and 51.7%, respectively. The time course of the continuation rates of CGRP-mAb is shown in Figure 2. Of the 146 patients who started CGRP-mAb treatment, 20 stopped treatment within three doses, with reasons including lack of efficacy (*n* = 16), financial difficulties (*n* = 2), side effects (*n* = 1), and pregnancy planning (*n* = 1). Thus, 126 patients continued CGRP-mAb treatment for three or more sessions. Of these 126 patients, 44 (34.9%) switched to another CGRP-mAb during the study period.

### 3.3. Withdrawal and Resumption Rates of CGRP-mAb Treatment

The results after discontinuation of CGRP-mAb treatment are shown in Table 3. A total of 146 patients received CGRP-mAb treatment, of whom 126 continued CGRP-mAb treatment for three or more doses. Of these 126 patients, 82 discontinued CGRP-mAb treatment at some point during the study period. The median [IQR] time of treatment discontinuation was 6.0 doses [5.0–9.0] after CGRP-mAb treatment initiation. Of the 82 patients who discontinued CGRP-mAb treatment, 63 patients wanted resumption due to headache recurrence. Therefore, the resumption rate was 76.8% (63/82). The median [IQR] interval from discontinuation to resumption was 2.0 months [1.0–3.0], with the breakdown being after 1 month (*n* = 30, 47.6%), 2 months (*n* = 10, 15.9%), and 3 months (*n* = 9, 14.3%). Thus, 77.8% (49/63) of patients resumed treatment within 3 months of discontinuing CGRP-mAb treatment. The remaining 14 patients (22.2%) resumed treatment after 4 months or more: after 4–6 months (*n* = 8) or 7 months or more (*n* = 6). In patients who resumed CGRP-mAb treatment, HIT-6 and MSQ scores decreased from 60.0 [56.3–64.3] and 29.0 [23.0–38.3] before resumption to 52.0 [48.0–60.0] and 21.0 [17.0–28.3] one month after resumption, respectively. Of the 82 patients who discontinued CGRP-mAb treatment, 19 did not resume treatment. Two patients (2.4%) had a follow-up period of less than 3 months after discontinuation, so the decision whether to resume or withdraw was undetermined. The remaining 17 patients did not resume CGRP-mAb treatment because there was no recurrence of headache symptoms. Therefore, the withdrawal rate was 20.7% (17/82).

### 3.4. Changes in HIT-6 and MSQ Scores

**Figure 3:**
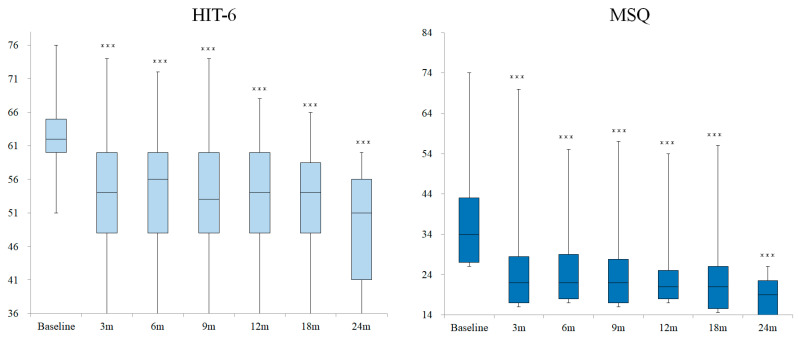
Changes in HIT-6 and MSQ scores in patients who continued receiving CGRP-mAb treatment. The boxes represent the 25% and 75% interquartile ranges, and the line inside the box represents the median. ***: Significant differences compared with baseline, *p* < 0.001, m: months.

The changes in HIT-6 and MSQ scores in patients who continued receiving CGRP-mAb treatment are shown in Figure 3. The median [IQR] HIT-6 scores at baseline and 3, 6, 9, 12, 18, and 24 months after CGRP-mAb treatment were 62.0 [60.0–65.0], 54.0 [48.0–60.0], 56.0 [48.0–60.0], 53.0 [48.0–60.0], 54.0 [48.0–60.0], 54.0 [48.0–58.5], and 51.0 [41.0–56.0], respectively. The median [IQR] MSQ scores at baseline and 3, 6, 9, 12, 18, and 24 months after CGRP-mAb treatment were 34.0 [27.0–43.0], 22.0 [17.0–28.5], 22.0 [18.0–29.0], 22.0 [17.0–27.8], 21.0 [18.0–25.0], 21.0 [15.5–26.0], and 19.0 [14.0–22.5], respectively. The HIT-6 and MSQ scores were significantly decreased from baseline at all time points (*p* < 0.001).

### 3.5. Comparison of Resumption and Withdrawal Groups

The results of the comparison between the resumption and withdrawal groups are shown in Table 4. There were no significant differences in background factors between the two groups. Although no significant differences were demonstrated, differences were suggested for two points: classification of migraine and timings of initiation of CGRP-mAb treatment. Specifically, for the classification of migraine, the resumption group and the withdrawal group had different rates of EM (19.0% vs. 35.3%) and of migraine with MOH (42.9% vs. 17.6%), respectively. Regarding timings of initiation of CGRP-mAb, the resumption group and the withdrawal group had different rates of early introduction (39.7% vs. 58.8%) and of late introduction (60.3% vs. 41.2%), respectively.

## 4. Discussion

In the present study, continuation and resumption rates of CGRP-mAb treatment for migraine were analyzed under real-world clinical conditions in which patients were free to choose own treatment. The analysis included 146 migraine patients with at least 3 months of follow-up after first initiating CGRP-mAb treatment. The continuation rate of CGRP-mAb treatment 12 months after treatment initiation was 58.8% (47/80). In the 82 patients who discontinued CGRP-mAb treatment due to its efficacy, the resumption rate was 76.8% (63/82), and the withdrawal rate was 20.7% (17/82). The interval from discontinuation to resumption of CGRP-mAb treatment was within 3 months in 77.8% (49/63) of patients.

This study is the first to report the time course of continuation rates of CGRP-mAb treatment under real-world conditions. The continuation rate of CGRP-mAb decreased linearly over time, but it was found to be 58.8% after 12 months and remained at 51.7% even after 24 months. Kang et al. reported one-year compliance of 140 migraine patients receiving CGRP mAbs in a real-world setting [19]. Fifty-nine patients were excluded, leaving 81 patients with a 12-month follow-up period for analysis. Forty patients (49.4%) continued CGRP-mAb treatment for 12 months after initiation, whereas 11 patients (13.6%) discontinued treatment due to symptom improvement. The report is consistent with the finding that approximately half of patients continued treatment with CGRP-mAb treatment 12 months after initiation. It should be noted that the continuation rate of CGRP-mAb treatment varies depending on the definition of continuation. In the present study, continuation included patients who discontinued temporarily or switched from CGRP-mAbs, which may explain why the continuation rate was slightly higher than that reported above. The HIT-6 and MSQ scores are direct indicators of the state of migraine. In the present study, significant improvements in both scores were maintained throughout the study period, demonstrating the effectiveness of CGRP-mAb treatment. Under real-world clinical conditions in which patients are free to choose their own treatment, patients will want to continue treatment if they experience benefit, so the continuation rate of CGRP-mAb treatment may be an indirect indicator of the effectiveness of the treatment.

This study showed that, when CGRP-mAb treatment was discontinued due to its effectiveness, the resumption rate was high (76.8%). An additional interesting finding was that 47.6% of patients who resumed treatment did so 1 month after discontinuation, and 77.8% did so by 3 months. Praeter et al. reported headache recurrence after a mandatory treatment holiday of 3 months in 243 patients receiving CGRP-mAbs [20]. Worsening headache symptoms occurred in 86% of patients, and the time to recurrence after discontinuation was 1 month in 59% and 3 months in 94%. In another report, 3 months after discontinuing CGRP-mAb treatment, the monthly migraine and headache days returned to the same level as before treatment initiation, and resumption of CGRP-mAbs reduced them to the same extent as initial treatment [21]. The recurrence rate and timing of headache exacerbations after discontinuation were similar whether CGRP-mAb treatment discontinuation was mandatory or voluntarily. Based on the results of these reports and the present study, 3 months after discontinuation of CGRP-mAb may be an important time point to determine whether to resume treatment. In the present analysis, patients who had discontinued CGRP-mAb treatment for less than 3 months were left undetermined as to whether they had resumed or were able to withdraw treatment. The withdrawal rate from CGRP-mAb treatment in the present study was found to be very low, at approximately 20%. However, because some patients resumed treatment more than 4 months after discontinuation, the withdrawal rate may decrease with a longer follow-up period after discontinuation.

No differences in background factors between the resumption and withdrawal groups in patients who discontinued CGRP-mAb treatment after it was effective were found. This result may indicate that there was truly no difference between the two groups, but it is also possible that the small number of cases in the withdrawal group prevented the identification of background factors. Interesting results were that the resumption group had higher rates of migraine with MOH (42.9% vs. 17.6%) and late introduction of CGRP-mAb (60.3% vs. 41.2%), whereas the withdrawal group had higher rates of EM (35.3% vs. 19.0%) and early introduction (58.8% vs. 39.7%). These results may suggest that early introduction of CGRP-mAb treatment in patients with mild headaches may increase the likelihood of treatment withdrawal. Although there are few reports of factors leading to CGRP-mAb treatment withdrawal, there are reports of factors that predict treatment efficacy [24,25,26]. A meta-analysis reported that predictors of CGRP-mAb efficacy were good response to triptans and unilateral pain with or without unilateral autonomic symptoms. Conversely, poor predictors were obesity, interictal allodynia, the presence of daily headaches, a higher number of unsuccessful previous prophylactic medications, and psychiatric comorbidities [24]. Pons-Fuster et al. reported that predictors of efficacy were no MOH and fewer basal monthly migraine and headache days [25]. A large, real-world study provided evidence that higher migraine frequency and baseline disability were associated with a lower likelihood of response to CGRP-mAbs, and earlier treatment initiation was recommended to increase the chances of treatment success [26]. These factors coincide with the differences in patient background factors between the resumption and withdrawal groups in the present study. Although this is speculative, it is possible that predictors of treatment response and factors leading to treatment withdrawal are related.

It is recognized that patients who progress to CM exhibit molecular, neuroimaging, and neurophysiological changes that may be associated with MOH, non-response to treatment, and failure to fully recovery with preventative treatment. Therefore, early initiation of effective and tolerable guideline-based preventive therapies has recently been recommended [27]. Furthermore, because CGRP-mAb treatment responds less well in patients with higher migraine frequency and greater baseline disability, early introduction of CGRP-mAbs is also important before migraine progression to CM or MOH [26]. According to a questionnaire survey, the prevalence of MOH in Japan was 2.32%, and it was more common among females and the middle-aged [28]. Additionally, the persistence rate of oral prophylactic medications declined from 49.7% at 3 months to 21.7% at 12 months in Japan. Similarly, the persistence rate of CGRP-mAb treatment declined from 85.6% at 3 months to 36.5% at 12 months [29]. These results suggest that preventive treatment for migraine patients remain challenging not only in early introduction but also continuation, and that there are substantial unmet needs for preventive treatment. There are various patterns in the time to onset of CGRP-mAb efficacy, including ultra-late responders and early responders [29,30]. Recent advances in the artificial intelligence technology have made it possible to predict the effectiveness of CGRP-mAb, allowing for the administration of CGRP-mAb at the appropriate time to patients who are likely to respond well to treatment, while avoiding unnecessary administration to patients predicted to have a poor response [31,32,33].

In this study, we used original definitions of continuation, resumption, and withdrawal of CGRP-mAbs. The main reason was that in real-world clinical practice, some patients experienced significant improvement in headache symptoms after CGRP-mAb administrations and wished to discontinue the administration. In other words, the CGRP-mAb was discontinued not because it was ineffective, but because it was highly effective. Reasons why patients may wish to discontinue CGRP-mAb despite its therapeutic benefits include high cost, reluctance to inject, and anxiety about continuing to receive injectable medication. Therefore, the definition of continuation included not only the continuous administration of CGRP-mAb, but also cases in which a patient experienced significant benefit from CGRP-mAb and wanted to temporarily discontinue administration, and then resumed administration after a short period. Because the majority of headache recurrences occurred within three months of discontinuation, we defined a short period as within three months. Conversely, we defined withdrawal as not re-administering CGRP-mAb even three months after discontinuation. Additionally, because a small number of patients experienced a recurrence of headache symptoms more than three months after discontinuation, Resumption was defined as when CGRP-mAb was resumed within 12 months of discontinuation.

The present study has limitations that should be noted. First, this was a single-center, retrospective, small case series, and it did not demonstrate the effectiveness of CGRP-mAb treatment compared with a control group. Second, the three CGRP-mAbs were not randomly assigned, but they were selected clinically by the physician and patients. Third, criteria were not set for the timing of starting CGRP-mAb treatment. Fourth, the choice to continue, discontinue, or resume CGRP-mAb treatment was based on patient preference without clear criteria, so the results of these treatment choices may be biased. Fifth, the continuation and resumption rates can vary depending on their definitions. The definition of continuation in this study was non-standard, which may lead to classification bias and an overestimation of continuation rate. Sixth, the withdrawal rate can vary depending on the follow-up period after discontinuation. Seventh, the use of rescue medications may affect the continuation or resumption of CGRP-mAbs, but this relationship was not analyzed. Finally, withdrawal group was compared with resumption group, but statistical power was limited due to the small number of cases.

## 5. Conclusions

Under real-world clinical conditions in which patients were free to choose their own treatment, the continuation rate of CGRP-mAb treatment at 12 months after treatment initiation was 58.8%, and more than half of patients were still on treatment at 24 months. In patients who discontinued CGRP-mAb treatment due to its efficacy, the resumption rate was 76.8%, and the withdrawal rate was 20.7%. The interval from discontinuation to resumption of CGRP-mAb treatment was within 3 months in 77.8% of patients. The continuation rates of CGRP-mAb treatment may be an indirect indicator of the effectiveness of the treatment. Three months after discontinuation of CGRP-mAb treatment may be an important time point to determine whether to resume treatment.

## Figures and Tables

**Table 1:** Demographic and clinical characteristics of the patients included in the analysis.

Characteristics	*n* = 146
Age (years), mean ± SD	36.8 ± 12.8
Sex, female; *n* (%)	122 (83.6%)
Onset years; *n* (%)	
Teens and younger	88 (60.3%)
20s	44 (30.1%)
30s	13 (8.9%)
40s and older	1 (0.7%)
Family history of headaches; *n* (%)	95 (65.1%)
History of psychiatric disorders; *n* (%)	20 (13.7%)
Migraine with aura; *n* (%)	70 (47.9%)
Smoking	7 (4.8%)
Precipitating factors	
Weather	109 (74.7%)
Temperature	47 (32.2%)
Alcohol	21 (14.4%)
Menstruation	60 (41.1%)
Classification of migraine	
Episodic migraine	31 (21.2%)
High frequency episodic migraine	37 (25.3%)
Chronic migraine	26 (17.8%)
Migraine with MOH	52 (35.6%)
Headache questionnaire scores	
HIT-6	62.0 [60.0–65.0]
MSQ	34.0 [27.0–43.0]
Types of oral prophylactic medications *	
Anticonvulsants	132 (90.4%)
Antidepressants	81 (55.5%)
Calcium channel blockers	59 (40.4%)
Beta blockers	7 (4.8%)
Initial CGRP-mAb	
Galcanezumab	55 (37.7%)
Fremanezumab	45 (30.8%)
Erenumab	46 (31.5%)
Timings of initiation of CGRP-mAb	
Early introduction	63 (43.2%)
Late introduction	83 (56.8%)

Data are expressed as median [interquartile ranges], CGRP-mAb: anti-calcitonin gene-related peptide monoclonal antibody, HIT-6: headache impact test-6, MOH: medication overuse headache, MSQ: migraine-specific quality of life questionnaire, *n*: number, SD: standard deviation, *: including overlaps.

**Table 2:** Breakdown of number of CGRP-mAb administered and number of switches.

	Number of Patients	Number of CGRP-mAb			
		GAL	FRE	ERE	Total
Total number of CGRP-mAb	173	766	558	463	1787
Number of CGRP-mAbs analyzed	146	730	545	450	1725
Continuation of initial CGRP-mAb	102	562	406	287	
Switch to 2nd CGRP-mAb	25	46	86	149	
Switch to 3rd CGRP-mAb	12	110	22	12	
Switch to 4th CGRP-mAb	7	12	31	2	
Number of CGRP-mAb excluded	27	36	13	13	62
Follow-up < 3 m	12	19	6	6	
Lost	12	12	6	7	
Non-naïve	3	5	1	0	
Time to first switch [IQR]	44	4 doses [2–7]	6 doses [5, 6]	4 doses [6–8]	6 doses [3–7]
Number of switches and reasons		25	19	26	70
Decreased efficacy		12	12	25	
Side efffects		4	7	0	
Cost		6	0	0	
Others		3	0	1	

CGRP-mAb: anti-calcitonin gene-related peptide monoclonal antibody, ERE: erenumab, FRE: fremanezumab, GAL: galcanezumab, IQR: interquartile ranges, m: months, 2nd: second, 3rd: third, 4th: fourth.

**Table 3:** Resumption and withdrawal rates of CGRP-mAb treatment.

Total patients receiving CGRP-mAb	146
Continued CGRP-mAb after the third dose	126
Attempted to discontinue CGRP-mAb	82
Median time to discontinuation	6.0 doses [5.0–9.0]
Resumption rate	76.8% (63/82)
Median interval from discontinuation to resumption	2.0 months [1.0–3.0]
1 month	47.6% (30/63)
2 months	15.9% (10/63)
3 months	14.3% (9/63)
4 months or more	22.2% (14/63)
Undetermined (follow-up < 3 months)	2.4% (2/82)
Withdrawal rate	20.7% (17/82)

CGRP-mAb: anti-calcitonin gene-related peptide monoclonal antibody.

**Table 4:** Comparison of resumption and withdrawal groups.

	Resumption Group	Withdrawal Group	*p*-Value	Statistical Power
	*n* = 63	*n* = 17		
Age (years), mean ± SD	40.3 ± 11.9	37.8 ± 12.6	0.499	0.119
Sex, female; *n* (%)	55 (87.3%)	13 (76.5%)	0.271	0.199
Onset years; *n* (%)			1.000	0.083
Teens and younger	36 (57.1%)	10 (58.8%)		
20s	20 (31.7%)	6 (35.3%)		
30s	6 (9.5%)	1 (5.9%)		
40s and older	1 (1.6%)	0 (0%)		
Family history of headaches; *n* (%)	42 (66.7%)	12 (70.6%)	1.000	0.056
History of psychiatric disorders; *n* (%)	7 (11.1%)	0 (0%)	0.336	0.317
Migraine with aura; *n* (%)	37 (58.7%)	7 (41.2%)	0.176	0.306
Smoking	4 (6.3%)	0 (0%)	0.571	0.195
Precipitating factors				
Weather	47 (74.6%)	12 (70.6%)	0.750	0.086
Temperature	23 (36.5%)	4 (23.5%)	0.390	0.196
Alcohol	11 (17.5%)	1 (5.9%)	0.446	0.238
Menstruation	27 (42.9%)	4 (23.5%)	0.164	0.346
Classification of migraine			0.204	0.371
Episodic migraine	12 (19.0%)	6 (35.3%)		
High frequency episodic migraine	13 (20.6%)	4 (23.5%)		
Chronic migraine	11 (17.5%)	4 (23.5%)		
Migraine with MOH	27 (42.9%)	3 (17.6%)		
Headache questionnaire scores				
HIT-6 (before)	62.0 [66.0–67.0]	62.0 [60.0–67.0]	0.777	0.119
HIT-6 (after 3 months)	52.0 [46.5–59.0]	50.0 [48.0–54.0]	0.487	0.061
MSQ (before)	35.0 [25.5–44.0]	35.0 [29.0–38.0]	0.967	0.055
MSQ (after 3 months)	21.0 [16.0–27.5]	21.0 [17.0–26.0]	0.967	0.106
Types of oral prophylactic medications *				
Anticonvulsants	53 (84.1%)	14 (82.4%)	1.000	0.054
Antidepressants	28 (44.4%)	7 (41.2%)	1.000	0.057
Calcium channel blockers	18 (28.6%)	2 (11.8%)	0.214	0.295
Beta blockers	4 (6.3%)	0 (0%)	1.000	0.051
Initial CGRP-mAb			0.743	0.091
Galcanezumab	23 (36.5%)	5 (29.4%)		
Fremanezumab	23 (36.5%)	6 (35.3%)		
Erenumab	17 (27.0%)	6 (35.3%)		
Timings of initiation of CGRP-mAb			0.179	0.292
Early introduction	25 (39.7%)	10 (58.8%)		
Late introduction	38 (60.3%)	7 (41.2%)		

Data are expressed as median [interquartile ranges], CGRP-mAb: anti-calcitonin gene-related peptide monoclonal antibody, HIT-6: headache impact test-6, MOH: medication overuse headache, MSQ: migraine-specific quality of life questionnaire, *n*: number, SD: standard deviation, *: including overlaps.

## Data Availability

The datasets used and/or analyzed during the current study are available from the corresponding author upon request.

## Abbreviations

The following abbreviations are used in this manuscript:

CM	chronic migraine
CGRP-mAb	anti-calcitonin gene-related peptide monoclonal antibody
EM	episodic migraine
ERE	erenumab
FRE	fremanezumab
GAL	galcanezumab
HFEM	high frequency episodic migraine
HIT-6	headache impact test-6
IQR	interquartile range
MOH	medication overuse headache
MSQ	migraine-specific quality of life questionnaire

## References

[1] Ashina M., Katsarava Z., Do T.P., Buse D.C., Pozo-Rosich P., Özge A., Krymchantowski A.V., Lebedeva E.R., Ravishankar K., Yu S. (2021). Migraine: Epidemiology and systems of care. Lancet.

[2] Raggi A., Leonardi M., Arruda M., Caponnetto V., Castaldo M., Coppola G., Della Pietra A., Fan X., Garcia-Azorin D., Gazerani P. (2024). Hallmarks of primary headache: Part 1—Migraine. J. Headache Pain.

[3] Shimizu T., Sakai F., Miyake H., Sone T., Sato M., Tanabe S., Azuma Y., Dodick D.W. (2021). Disability, quality of life, productivity impairment and employer costs of migraine in the workplace. J. Headache Pain.

[4] GBD 2016 Disease and Injury Incidence and Prevalence Collaborators (2017). Global, regional, and national incidence, prevalence, and years lived with disability for 328 diseases and injuries for 195 countries, 1990–2016: A systematic analysis for the Global Burden of Disease Study 2016. Lancet.

[5] Matsumori Y., Ueda K., Komori M., Zagar A.J., Kim Y., Jaffe D.H., Takeshima T., Hirata K. (2022). Burden of Migraine in Japan: Results of the ObserVational Survey of the Epidemiology, tReatment, and Care Of MigrainE (OVERCOME [Japan]) Study. Neurol. Ther..

[6] Tanei T., Yamamoto S., Maesawa S., Nishimura Y., Ishizaki T., Nagashima Y., Ito Y., Hashida M., Suzuki T., Hamasaki H. (2025). Identifying Factors Associated with the Efficacy of Lasmiditan 50 mg as an Acute Treatment for Migraine Attacks Under Various Dosing Conditions in Real-World Clinical Practice. Neurol. Int..

[7] Goadsby P.J., Reuter U., Hallström Y., Broessner G., Bonner J.H., Zhang F., Sapra S., Picard H., Mikol D.D., Lenz R.A. (2017). A Controlled Trial of Erenumab for Episodic Migraine. N. Engl. J. Med..

[8] Skljarevski V., Matharu M., Millen B.A., Ossipov M.H., Kim B.K., Yang J.Y. (2018). Efficacy and safety of galcanezumab for the prevention of episodic migraine: Results of the EVOLVE-2 Phase 3 randomized controlled clinical trial. Cephalalgia.

[9] Dodick D.W., Silberstein S.D., Bigal M.E., Yeung P.P., Goadsby P.J., Blankenbiller T., Grozinski-Wolff M., Yang R., Ma Y., Aycardi E. (2018). Effect of Fremanezumab Compared With Placebo for Prevention of Episodic Migraine: A Randomized Clinical Trial. JAMA.

[10] Sakai F., Suzuki N., Kim B.K., Tatsuoka Y., Imai N., Ning X., Ishida M., Nagano K., Iba K., Kondo H. (2021). Efficacy and safety of fremanezumab for episodic migraine prevention: Multicenter, randomized, double-blind, placebo-controlled, parallel-group trial in Japanese and Korean patients. Headache.

[11] Takeshima T., Sakai F., Hirata K., Imai N., Matsumori Y., Yoshida R., Peng C., Cheng S., Mikol D.D. (2021). Erenumab treatment for migraine prevention in Japanese patients: Efficacy and safety results from a phase 3, randomized, double-blind, placebo-controlled study. Headache.

[12] Versijpt J., Paemeleire K., Reuter U., MaassenVanDenBrink A. (2025). Calcitonin gene-related peptide-targeted therapy in migraine: Current role and future perspectives. Lancet.

[13] Tanei T., Fuse Y., Maesawa S., Nishimura Y., Ishizaki T., Nagashima Y., Mutoh M., Ito Y., Hashida M., Suzuki T. (2024). Real-world clinical results of CGRP monoclonal antibody treatment for medication overuse headache of migraine without abrupt drug discontinuation and no hospitalization. Heliyon.

[14] Hirata K., Ueda K., Komori M., Zagar A.J., Selzler K.J., Nelson A.M., Han Y., Jaffe D.H., Matsumori Y., Takeshima T. (2021). Comprehensive population-based survey of migraine in Japan: Results of the ObserVational Survey of the Epidemiology, tReatment, and Care Of MigrainE (OVERCOME [Japan]) study. Curr. Med. Res. Opin..

[15] Sacco S., Amin F.M., Ashina M., Bendtsen L., Deligianni C.I., Gil-Gouveia R., Katsarava Z., MaassenVanDenBrink A., Martelletti P., Mitsikostas D.D. (2022). European Headache Federation guideline on the use of monoclonal antibodies targeting the calcitonin gene related peptide pathway for migraine prevention—2022 update. J. Headache Pain.

[16] Al-Hassany L., Lyons H.S., Boucherie D.M., Farham F., Lange K.S., Marschollek K., Onan D., Pensato U., Storch E., Torrente A. (2023). European Headache Federation School of Advanced Studies (EHF-SAS). The sense of stopping migraine prophylaxis. J. Headache Pain.

[17] Rattanawong W., Rapoport A., Srikiatkhachorn A. (2022). Neurobiology of migraine progression. Neurobiol. Pain.

[18] Sacco S., Bendtsen L., Ashina M., Reuter U., Terwindt G., Mitsikostas D.D., Martelletti P. (2019). European headache federation guideline on the use of monoclonal antibodies acting on the calcitonin gene related peptide or its receptor for migraine prevention. J. Headache Pain.

[19] Kang M.K., Sohn J.H., Cha M.J., Kim Y.H., Hong Y., Im H.J., Cho S.J. (2025). One-Year Compliance After Calcitonin Gene-Related Peptide Monoclonal Antibody Therapy for Migraine Patients in a Real-World Setting: A Multicenter Cross-Sectional Study. J. Clin. Med..

[20] De Praeter R., Tuerlinckx E., Schoenen J., Boon E., Sava S.L., Debruyne F., Delmotte K., De Pauw A., Van Dycke A., Van Humbeeck C. (2025). Migraine treatment with CGRP monoclonal antibodies: Patient evaluation of a mandatory treatment holiday in Belgium. Acta Neurol. Belg..

[21] Raffaelli B., Terhart M., Mecklenburg J., Neeb L., Overeem L.H., Siebert A., Steinicke M., Reuter U. (2022). Resumption of migraine preventive treatment with CGRP(-receptor) antibodies after a 3-month drug holiday: A real-world experience. J. Headache Pain.

[22] International Headache Society (2018). Headache Classification Committee of the International Headache Society (IHS) The International Classification of Headache Disorders, 3rd edition. Cephalalgia.

[23] Kanda Y. (2013). Investigation of the freely available easy-to-use software ‘EZR’ for medical statistics. Bone Marrow Transpl..

[24] Hong J.B., Lange K.S., Overeem L.H., Triller P., Raffaelli B., Reuter U. (2023). A Scoping Review and Meta-Analysis of Anti-CGRP Monoclonal Antibodies: Predicting Response. Pharmaceuticals.

[25] Pons-Fuster E., Lozano-Caballero O., Martín-Balbuena S., Lucas-Ródenas C., Mancebo-González A., De Gorostiza-Frías I., González-Ponce C.M. (2024). Anti-CGRP monoclonal antibodies in resistant migraine: Preliminary real-world effectiveness and clinical predictors of response at two years. Int. J. Clin. Pharm..

[26] Caronna E., Gallardo V.J., Egeo G., Vázquez M.M., Castellanos C.N., Membrilla J.A., Vaghi G., Rodríguez-Montolio J., Fabregat Fabra N., Sánchez-Caballero F. (2024). Redefining migraine prevention: Early treatment with anti-CGRP monoclonal antibodies enhances response in the real world. J. Neurol. Neurosurg. Psychiatry.

[27] Pozo-Rosich P., Caronna E., Sacco S., Peres M.F.P., Ashina S., Özge A., Ahmed F., Velez-Jimenez M.K., Jenkins B., Wang S.J. (2025). Early treatment in migraine—A call to shift prevention from attacks to disease progression: A position statement from the International Headache Society. Cephalalgia.

[28] Katsuki M., Yamagishi C., Matsumori Y., Koh A., Kawamura S., Kashiwagi K., Kito T., Entani A., Yamamoto T., Ikeda T. (2022). Questionnaire-based survey on the prevalence of medication-overuse headache in Japanese one city-Itoigawa study. Neurol. Sci..

[29] Takeshima T., Ahmadyar G., Duan M., Yamazaki T., Inoue S., Nishimura C. (2025). Real world treatment patterns and unmet needs of migraine preventive treatments in Japan: JMDC claims analysis. Curr. Med. Res. Opin..

[30] Katsuki M., Kashiwagi K., Kawamura S., Tachikawa S., Koh A. (2023). One-Time Use of Galcanezumab or Fremanezumab for Migraine Prevention. Cureus.

[31] Chiang C.C., Schwedt T.J., Dumkrieger G., Wang L., Chao C.J., Ouellette H.A., Banerjee I., Chen Y.C., Jones B.M., Burke K.M. (2024). Advancing toward precision migraine treatment: Predicting responses to preventive medications with machine learning models based on patient and migraine features. Headache.

[32] Gonzalez-Martinez A., Pagán J., Sanz-García A., García-Azorín D., Rodríguez-Vico J.S., Jaimes A., García A.G., de Terán J.D., González-García N., Quintas S. (2022). Machine-learning-based approach for predicting response to anti-calcitonin gene-related peptide (CGRP) receptor or ligand antibody treatment in patients with migraine: A multicenter Spanish study. Eur. J. Neurol..

[33] Romozzi M., Lokhandwala A., Vollono C., Vigani G., Burgalassi A., García-Azorín D., Calabresi P., Chiarugi A., Geppetti P., Iannone L.F. (2024). An evolving machine-learning-based algorithm to early predict response to anti-CGRP monoclonal antibodies in patients with migraine. Cephalalgia.

